# Number processing outside awareness? Systematically testing sensitivities of direct and indirect measures of consciousness

**DOI:** 10.3758/s13414-021-02312-2

**Published:** 2021-05-10

**Authors:** Iris A. Zerweck, Chung-Shan Kao, Sascha Meyen, Catarina Amado, Martin von Eltz, Maren Klimm, Volker H. Franz

**Affiliations:** 1 Department of Computer Science, Experimental Cognitive Science, University of Tübingen, Sand 6, Tübingen, 72076 Germany; 2 General Psychology, Universität Hamburg, Hamburg, Germany

**Keywords:** Priming, Visual perception, Signal detection theory

## Abstract

In priming research, it is often argued that humans can discriminate stimuli outside consciousness. For example, the semantic meaning of numbers can be processed even when the numbers are so strongly masked that participants are not aware of them. These claims are typically based on a certain pattern of results: *Direct measures* indicate no conscious awareness of the masked stimuli, while *indirect measures* show clear priming effects of the same stimuli on reaction times or neurophysiological measures. From this pattern, preserved (unconscious) processing in the indirect task is concluded. However, this widely used *standard reasoning* is problematic and leads to spurious claims of unconscious processing. Such problems can be avoided by comparing sensitivities of direct and indirect measures. Many studies are affected by these problems, such that a reassessment of the literature is needed. Here, we investigated whether numbers can be processed unconsciously. In three experiments, we replicated and extended well-established effects of number priming over a wide range of stimulus visibilities. We then compared the standard reasoning to a sensitivity analysis, where direct and indirect effects are compared using the same metric. Results show that the sensitivities of indirect measures did not exceed those of direct measures, thereby indicating no evidence for preserved unconscious processing when awareness of the stimuli is low. Instead, it seems that at low visibility there is residual processing that affects direct and indirect measures to a similar degree. This suggests that similar processing modes cause those effects in direct and indirect measures.

## Introduction

In the past decades, research on unconscious priming has received increasing attention. One important claim here is that unconscious processing can be better than conscious processing, even for complex cognitive tasks (Dehaene et al., 1998; Pessiglione et al., 2007; ten Brinke et al., 2014; Wójcik et al., 2019). To establish this, researchers typically use the masked priming paradigm, where participants perform two tasks (“direct” and “indirect” tasks) and researchers attempt to compare the task performances of both tasks (Dehaene et al., 1998; Draine & Greenwald, 1998; Finkbeiner, 2011; Mattler, 2003; Peremen & Lamy, 2014; Reingold & Merikle, 1988; Wang et al., 2017). In the direct task, participants directly classify a masked stimulus (“prime”) and perform close to chance. In the indirect task, participants respond to a “target” stimulus following the masked prime and the prime has clear and significant effects on reaction times (RTs) or brain activity (EEG, fMRI). From such a pattern of significant priming effects in the indirect task and close-to-chance performance in the direct task, researchers typically infer that there is better sensitivity to the stimulus categories of the prime in the indirect than in the direct task. Further, because the close-to-chance sensitivity in the direct task is interpreted as poor conscious processing of the prime, the priming effect in the indirect task is assumed to be (mainly) due to unconscious processing, suggesting qualitatively different conscious versus unconscious processing modes (Hannula et al., 2005; Lamme & Roelfsema, 2000; J. S. Morris et al., 1998; ten Brinke et al., 2014). This rationale is referred to as the *standard reasoning* here. It is widely applied to unconscious priming research and the results derived from such studies have a strong impact on current theories about unconscious processing (Dehaene & Changeux, 2011; Hassin, 2013; Kouider & Dehaene, 2007; ten Brinke et al., 2016).

Importantly, the standard reasoning is inadvisable for several reasons. Let us sketch the two most important ones. First, the direct task is often severely underpowered, such that a non-significant close-to-chance performance does not necessarily imply absence of awareness (Vadillo et al., 2016).

Second, it has been shown that a clear priming effect does not imply good sensitivity for the prime. Franz and von Luxburg (2015) reanalyzed the data of a study that concluded better unconscious (indirect) as compared to conscious (direct) sensitivity based on the standard reasoning (ten Brinke et al., 2014). Instead of conducting two separate significance tests in the direct and indirect tasks and then using a pattern of significant (indirect) versus non-significant (direct) effects, as is common practice in the standard reasoning, Franz and von Luxburg (2015) argue that it is mandatory to determine sensitivities to the prime in both tasks (similar arguments have been raised by Eriksen, 1960, and Reingold & Merikle, 1988). By transforming performances in both tasks into measures of sensitivity and then comparing those sensitivities, Franz and von Luxburg (2015) found that the sensitivity in the indirect task was just as poor as in the direct task, therefore indicating no evidence for better unconscious than conscious processing of the prime in the indirect task of ten Brinke et al. (2014). This critique has led to the general question of whether there are further erroneous claims of unconscious processing in the priming literature and how to detect them.

Following this critique, our workgroup has reanalyzed 15 highly influential studies in the field of unconscious priming (Meyen et al., in press) and found that in most studies the sensitivity to the prime in the direct task was not different from the sensitivity in the indirect task, while the large majority seems to have mistakenly concluded a better indirect as compared to a direct task sensitivity.

Taken together, there is strong evidence that many claims of better unconscious than conscious processing are problematic and the question arises under which circumstances a better sensitivity in the indirect as compared to the direct task can be concluded. In line with Meyen et al. (in press), we will use the term indirect task advantage (ITA) for a situation with a better indirect task sensitivity to the prime as compared to a direct task sensitivity.

In the following sections, we first describe the problematic standard reasoning and the more appropriate sensitivity analysis. Then, we describe the present study, where we examined the evidence for an ITA in the important case of number priming. We conducted three experiments using the stimuli of a highly influential landmark study (Dehaene et al., 1998), and corresponding replications (Kouider & Dehaene, 2009; Naccache & Dehaene, 2001a), which we together refer to as the “original studies” in the following. We used a similar paradigm to that used in the original studies, and compared the results of the standard reasoning to the sensitivity analysis (see below). We also show that the sensitivity analysis does not support better sensitivity to the prime in the indirect task as compared to the direct task and suggest that researchers should indeed be cautious about interpreting typical priming effects as evidence for a good sensitivity.

## Response-priming paradigm and the fallacy of the standard reasoning

We first describe the standard reasoning used in the response-priming paradigm (Finkbeiner, 2011; Lamy et al., 2009; Ortells et al., 2016; Pessiglione et al., 2007; Van den Bussche et al., 2009) and why the standard reasoning is misleading. In a typical experiment, a masked “prime” stimulus is followed by a “target” stimulus, and participants perform two tasks: In the direct task, researchers want to establish that participants are not consciously aware of the masked prime. For example, Naccache and Dehaene (2001a) used numbers as prime and target stimuli and participants judged in the direct task whether the *prime* was larger or smaller than 5. Typically, participants are close to chance (close-to-zero sensitivity) in the direct task. This result is interpreted as evidence for poor conscious perception of the prime.

In the indirect task, researchers want to establish that there are nevertheless indirect effects of the prime. For example, the participants of Naccache and Dehaene (2001a) decided in the indirect task whether the *target* was larger or smaller than 5. Typically, trials with congruent prime and target (e.g., prime and target both larger than 5) show faster RTs than trials with incongruent prime and target (e.g., prime larger than 5 and target smaller than 5). This result is interpreted as evidence that the prime is processed and (because of the close-to-zero sensitivity in the direct task) that it is processed outside consciousness. For example, Dehaene et al. (1998) argued that participants “could neither reliably report [the prime’s] presence or absence nor discriminate it from a nonsense string [...]. Nevertheless, [based on the priming effects] we show here that the prime is processed to a high cognitive level” (p. 597).

**Fig. 1:**
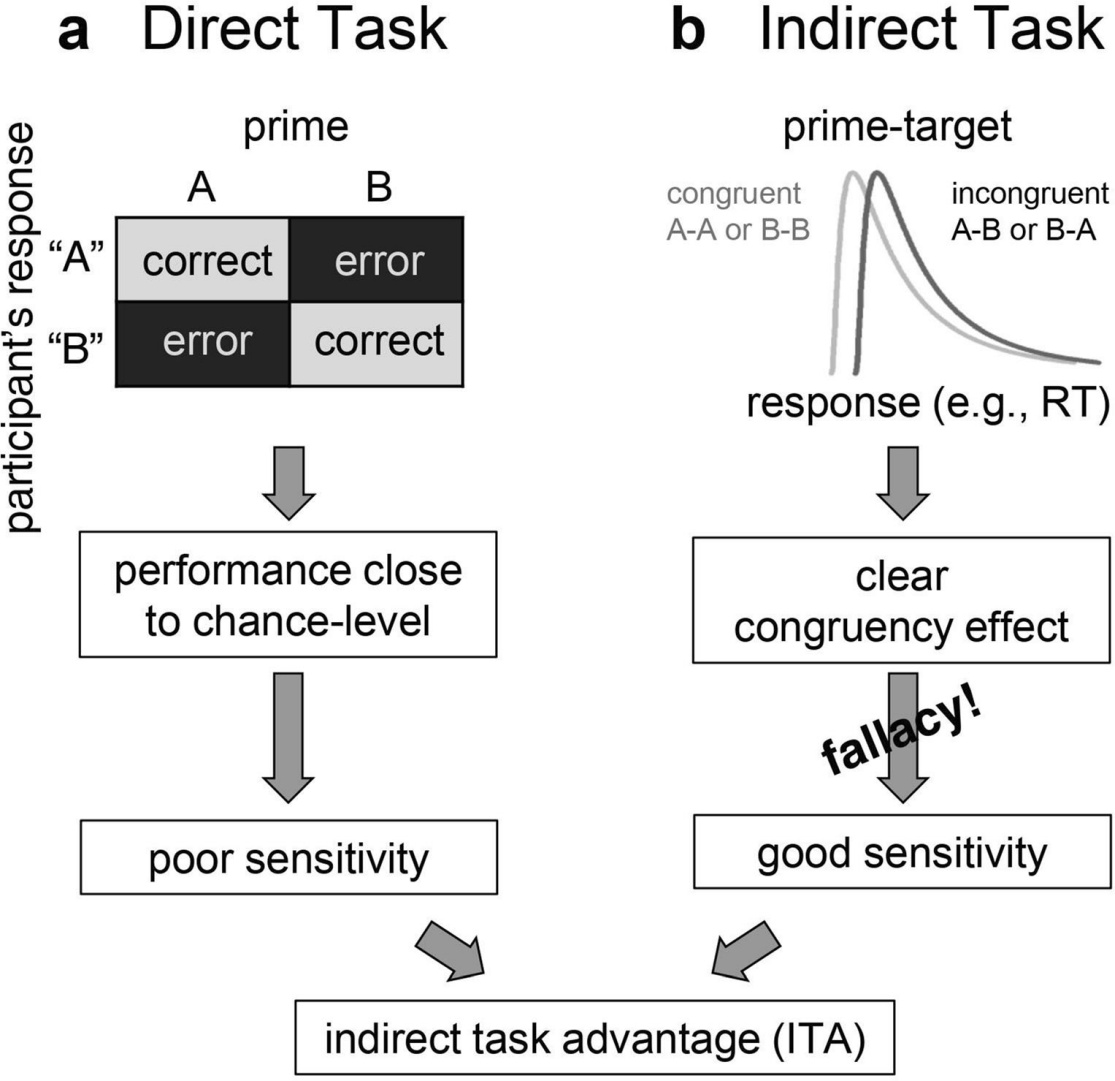
Step 1 of the standard reasoning to infer a better indirect task performance (ITA) as compared to a direct task performance. (**a**) In the direct task, participants classify a masked prime stimulus as belonging to category A or B (e.g., A = smaller than 5, B = larger than 5). Based on the rates of hits and false alarms, the percentage of correctly classified primes is then calculated and compared to chance level (50%). Typically, responses display close-to-zero sensitivity here, which is interpreted as poor sensitivity for the prime. (**b**) In the indirect task, participants respond to a target stimulus preceded by the masked prime. Typically, the prime has effects on reaction times (RTs). Trials with primes and targets belonging to the same category A-A or B-B (congruent, e.g., prime and target both larger than 5) show faster RTs than trials with primes and targets belonging to different categories A-B or B-A (incongruent, e.g., prime smaller but target larger than 5). This results in a significant priming effect. From this clear priming effect, the standard reasoning infers a relatively good sensitivity for the prime in the indirect task. However, there is a fallacy in interpreting a significant effect as being indicative of a good sensitivity (for details see our Fig. 2; Franz & von Luxburg, 2015, and Meyen et al., in press). Based on this fallacy, the standard reasoning infers from a clear priming effect in the indirect task on the one hand, and a poor classification performance in the direct task on the other hand, that participants’ performance is better on the indirect task compared to the direct task (ITA). One possibility for resolving this issue is to transform both tasks into the same metric (e.g., by calculating the sensitivity *d’*) and compare them directly with each other

Meyen et al. (in press) examined the standard reasoning and showed that it is based on two consecutive inferential steps: In Step 1, researchers want to establish that the sensitivity for the prime is relatively poor in the direct task, but relatively good in the indirect task. In the direct task, researchers infer from the close-to-zero sensitivity (*d’* close to zero) a poor sensitivity for the prime (Fig. 1a). In the indirect task, they infer from a significant priming effect a relatively good sensitivity for the prime and conclude a higher sensitivity for the prime in the indirect task as compared to the direct task. That is, they infer an ITA (Fig. 1b).

This ITA in Step 1 is a prerequisite for Step 2, in which the direct task is seen as a measure for (mainly) conscious processes and the indirect task is seen as a measure for (mainly) unconscious processes. The ITA is then interpreted as evidence for unconscious processing of the prime.

However, there is a fallacy in Step 1 of the standard reasoning: A clear (and significant) priming effect in the indirect task does not imply good sensitivity for the prime. The clear priming effect can be consistent with a very bad as well as with a very good underlying sensitivity.

**Fig. 2:**
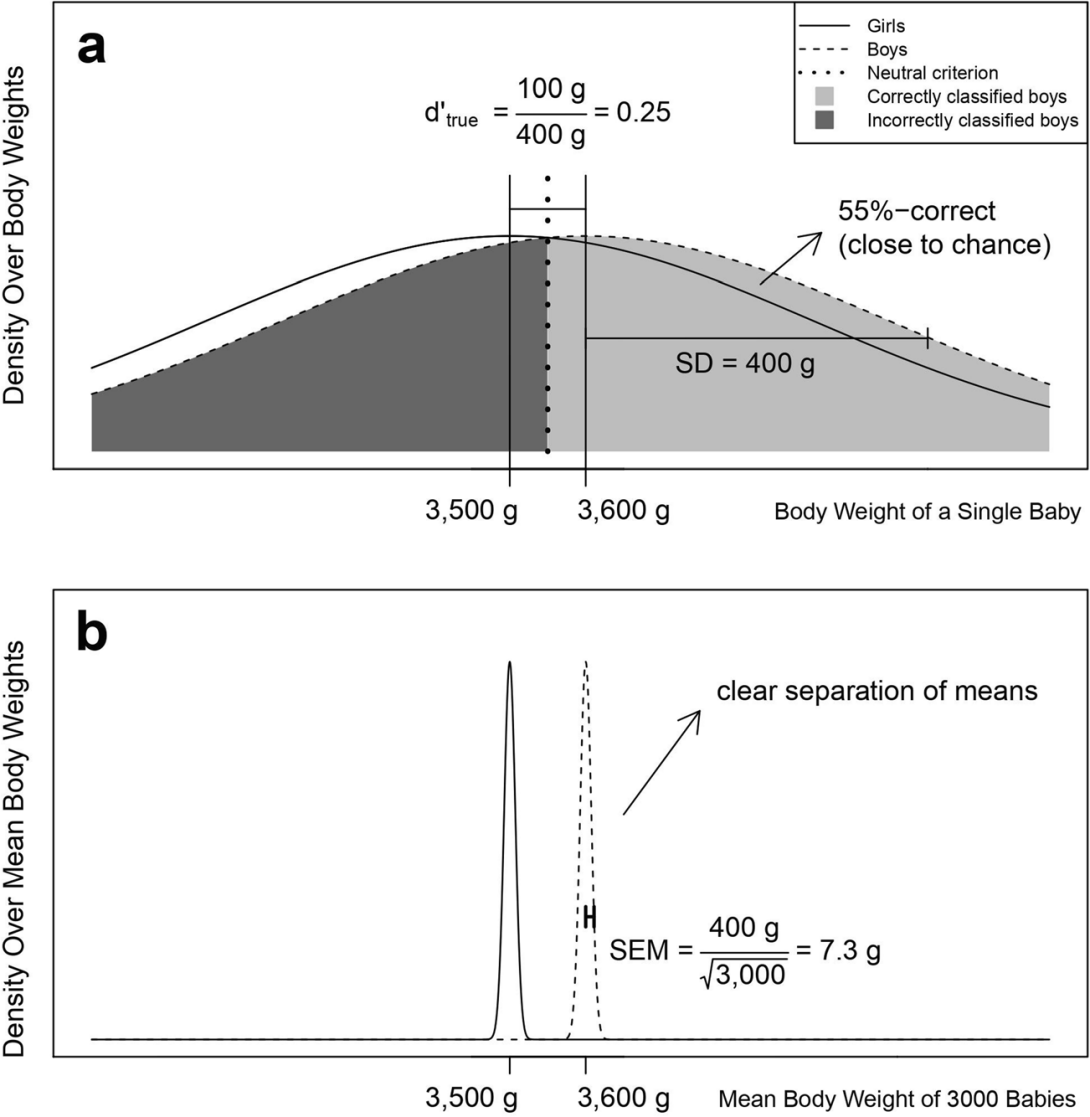
Toy-example. We show why the standard reasoning is problematic: A significant priming effect does not necessarily imply good sensitivity (or classification) of the prime. Consider a group of babies. (**a**) The mean birth weight of baby boys is usually greater than the mean birth weight of baby girls (approximately 100 g). If you want to classify individual babies as girls or boys based on their birth weights, your accuracy would be poor due to a large overlap of the weight distributions (reflected by the poor sensitivity of d’ = 0.25 and a poor classification performance of 55% correct). (**b**) Now consider you took two groups of, say, 3,000 baby boys and 3,000 baby girls. A standard significance test would show that these groups differ in birth weight, which corresponds to the priming effect in the standard paradigm. This demonstrates that a significant effect can coexist with a relatively poor sensitivity (or classification performance). Therefore, a significant priming effect does not imply good sensitivity. Figure obtained with permission from Meyen et al. (in press)

To further illustrate this, consider a simple example, where exactly the same information is the basis for the performance in hypothetical “direct” and “indirect” tasks. Think of a group of babies. Newborn boys weigh on average slightly more than newborn girls (Fig. 2). If we wanted to classify the sex of individual babies based on their weight, our classification performance would be poor due to a large overlap of the weight distributions (Fig. 2a). This corresponds to what is typically measured in the direct task. That is, how well participants classify the prime (e.g., whether it is a number larger or smaller than 5) and it is typically found that participants show poor performance (just as we show poor performance when classifying a baby’s sex based on the baby’s weight).

Now consider you took two groups of, say, 3,000 baby boys and 3,000 baby girls. You will find that the mean weights of those two groups will clearly differ (Fig. 2b). This corresponds to what is typically measured in the indirect task: The mean RTs are calculated across a large number of congruent versus incongruent trials (often with something like 3,000 congruent and incongruent trials in a typical experiment). It is typically found that those mean RTs are clearly (and significantly) different (just as the mean group weights of our 3,000 baby boys and 3,000 baby girls will clearly differ).

Here is the catch: In the baby example, we know that the sensitivity of body weight to the babies’ sex is exactly the same in both tasks (because our hypothetical “direct” and “indirect” tasks are based on exactly the same weight distributions). Nevertheless, the standard reasoning would infer better sensitivity in the “indirect” task than in the “direct” task, which is incorrect. The problem arises because the standard reasoning asks the wrong question in the indirect task. The underlying statistical question when performing a t-test only asks: Is the expected value for one condition different from that for the other condition? But it does not answer the question of how much sensitivity there is for the stimulus. In short, our example shows that the standard reasoning is unsuitable and that a clear (and significant) priming effect does not imply good sensitivity in the indirect task (cf. Franz & von Luxburg, 2015 and Meyen et al., in press).

## The sensitivity analysis

The baby example demonstrates that a significant priming effect does not imply good sensitivity. To compare the direct and the indirect task performances, the indirect task needs to be transformed into the same metric as the direct task (e.g., % correct or sensitivity *d’*), as has been argued before (Reingold & Merikle, 1988).

One approach is to apply a technique proposed by Franz and von Luxburg (2015): a median-split of the RTs, which is valid for the typical balanced designs with an equal number of congruent and incongruent trials (Franz & von Luxburg, 2015; Meyen et al., in press). For the median-split-technique, we take for each participant all RTs independent of the condition and calculate the median. For the next step, we accept that participants respond faster on congruent trials than on incongruent trials (which we know from the priming effect). Trials with RTs shorter than the median are classified as congruent and trials with RTs longer than the median are classified as incongruent. Accordingly, congruent trials with RTs shorter than the median are counted as hits and those with RTs longer than the median are counted as misses (conversely for incongruent RTs). Based on this, we calculate the percentage of correctly classified trials in the indirect task and compare them to the direct task. Additionally, we calculate the sensitivity *d’* derived from Signal Detection Theory for the direct and the indirect task using the formula *d’* = Φ^(-1) (HR)-Φ^(-1) (FA), with Φ^(-1) being the inverse normal CDF, HR the hit rate, and FA the false alarms (Green & Swets, 1988). Please note, for the indirect task this formula is adequate even if the RT distributions are right-skewed, because the distributional properties (such as HR and FA) from normal distributions stay constant when transformed to lognormal distributions (for technical proof see Meyen et al., in press). The technique has also been used – sporadically – before (e.g., Schmidt, 2002). The method is necessary to ensure that both tasks have equal measures such that a test for the difference can be conducted to compare them directly. For further details and discussions, see Franz and von Luxburg, (2015) and Meyen et al. (in press).

## Our study

For our experiments we used the stimulus and task settings of the highly influential study by Dehaene et al. (Dehaene et al., 1998; for a detailed review, see also Kouider & Dehaene, 2007) and the corresponding replications (Kouider & Dehaene, 2009; Naccache & Dehaene, 2001a). As mentioned above, we refer to these studies as the “original” studies. We used a similar paradigm to that used in the original studies, transformed the measures from both tasks into the same metric (sensitivity *d’*) to compare them directly, adapted the number of trials to equate the power in both tasks, and applied parametric variations of stimulus perceptibility (cf. F. Schmidt et al., 2011; T. Schmidt & Vorberg, 2006) to ensure that we do not miss the conditions with an ITA. The strength of parametric variations is that researchers can observe and compare the changes of the two measures over a wide range. We focused on the behavioral part of the original studies (Dehaene et al., 1998, also had conditions with EEG and fMRI), since most researchers in this field measure RTs in the indirect task.

In Experiment 1, we successfully replicated the behavioral findings of the original studies and compared the standard analysis to the sensitivity analysis. Overall, we found no evidence for an ITA. Next, we parametrically varied prime contrast (Experiment 2) and prime duration (Experiment 3) and again found no evidence for an ITA. Instead, we consistently found that the indirect task sensitivity is just as poor as the direct task sensitivity when prime visibility is low. If prime visibility is high, then the indirect task sensitivity is even lower than the direct task sensitivity, which is just the opposite of an ITA.

## Experiment 1

In this experiment, we aimed to replicate the behavioral findings of the original studies and then to apply the sensitivity analysis to the results, as suggested by Franz and von Luxburg (2015) and Meyen et al. (in press).

The direct tasks differed somewhat across the original studies: Dehaene et al. (1998) employed two direct tasks: (a) the prime was either present or absent, such that participants had to detect the prime (detection task), and (b) the prime was either a digit or a random string, which participants had to discriminate (discrimination task). Later, however, Naccache and Dehaene (2001a, p. 222) argued that another task is better suited for comparison to the indirect task: They kept the stimulus sequence constant for both the direct and the indirect task. Participants classified in the direct task the prime as being smaller or larger than 5 and in the indirect task the target as being smaller or larger than 5 (such a matching of the stimulus sequence of direct and indirect tasks was also advocated by Reingold & Merikle, 1988, and Schmidt & Vorberg, 2006). Subsequently, this task was also used by Kouider and Dehaene (2009) and also by us. Note that Kouider and Dehaene (2009) additionally tested different stimulus modalities which we did not. Thus, our experimental setup follows the task settings of Dehaene et al. (1998) for the indirect task and the task settings of Naccache and Dehaene (2001a) as well as those of Kouider and Dehaene (2009) for the direct task.

We expected to find similar behavioral results as in the original studies: In the direct task, we expected that participants are close to chance-level performance. In the indirect task, we expected a clear effect of prime-target congruency on RTs (i.e., a priming effect). We then tested whether the sensitivity in the indirect task exceeds the sensitivity in the direct task (ITA), as measured by the sensitivity index *d’*.

### Participants

Eighteen participants (14 female and four male) with normal or corrected-to-normal vision took part in Experiment 1. The participants were volunteers recruited from the student population of the University of Tübingen. They were fully naive with regard to the purpose of the study. Seventeen participants were right-handed by self-report. The age of the participants ranged from 20 to 30 years (*M*_age_ = 23.6, *SD*_age_ = 3.7). They received either course credits or a payment (app. 10 €) for their participation. The experiments were approved by the local ethics committee, and written consent was obtained from each participant before beginning the experiment.

#### Sample size and power analysis

To determine the sample size for our experiments, we optimized different aspects: (a) we wanted a large statistical power to find the typical pattern of results according to the standard reasoning, as well as any potentially interesting ITA in the sensitivity analysis. (b) the direct task should not be underpowered, and (c) the experiment should not be too long to tire the participants too much. To achieve these goals, we chose a relatively large sample size of N = 18 participants and K = 256 trials per participant and task (with direct and indirect task being allotted the same number of trials). This ensured a larger number of observations (N participants * K trials * 2 tasks = 18 * 256 * 2 = 9,216) than in any of the original studies (cf. Fig. 4). Note that standardized effect sizes do not account for the number of trials tested (Baguley, 2009; P. Morris, 2020). Therefore, we used our experiment in Meyen et al. (in press) to determine the to-be-expected RT effect and its variability. This resulted in a power of 99.74% for an RT difference of 12 ms (with SD = 11.83 ms, N = 18). The underlying power analysis to find an ITA was based on the following reasoning: We considered a sensitivity difference of *d’*_*indirect*_ *– d’*_*direct*_ = 0.25 to be the smallest interesting ITA effect (this corresponds to a neutral observer being, e.g., 50% correct in the direct task and 55% correct in the indirect task) and a sensitivity difference of ∆*d’* = 0.51 to be a theoretically more interesting ITA effect (corresponding to 50% correct in the direct task and 60% correct in the indirect task). Again, we used Meyen et al. (in press) to estimate the variability of these ITA effects. This resulted in a power of 91.10% for an ITA of ∆*d’* = 0.25 and a power of >99.9% for an ITA of ∆*d’* = 0.51.

### Setup

The experiment took place in a dimly lit, sound-attenuated audiometry cabin (audiometry test booth A:BOX, Desone Modulare Akustik, Berlin, Germany). Participants were seated in front of a ViewPixx monitor (VIEWPixx-3D, VPixx Technologies Inc., Canada; 1,920 x 1,200 pixels, 22.5 in. display size, 120 Hz) at a viewing distance of about 50 cm. RTs were recorded using a button press box (RESPONSEPixx Handheld, VPixx Technologies Inc., Canada). The experiment was programmed in MATLAB R2017b (9.3.0.713579) using Psychophysics Toolbox (3.0.14; Brainard, 1997; Kleiner, 2010).

### Stimuli

**Fig. 3:**
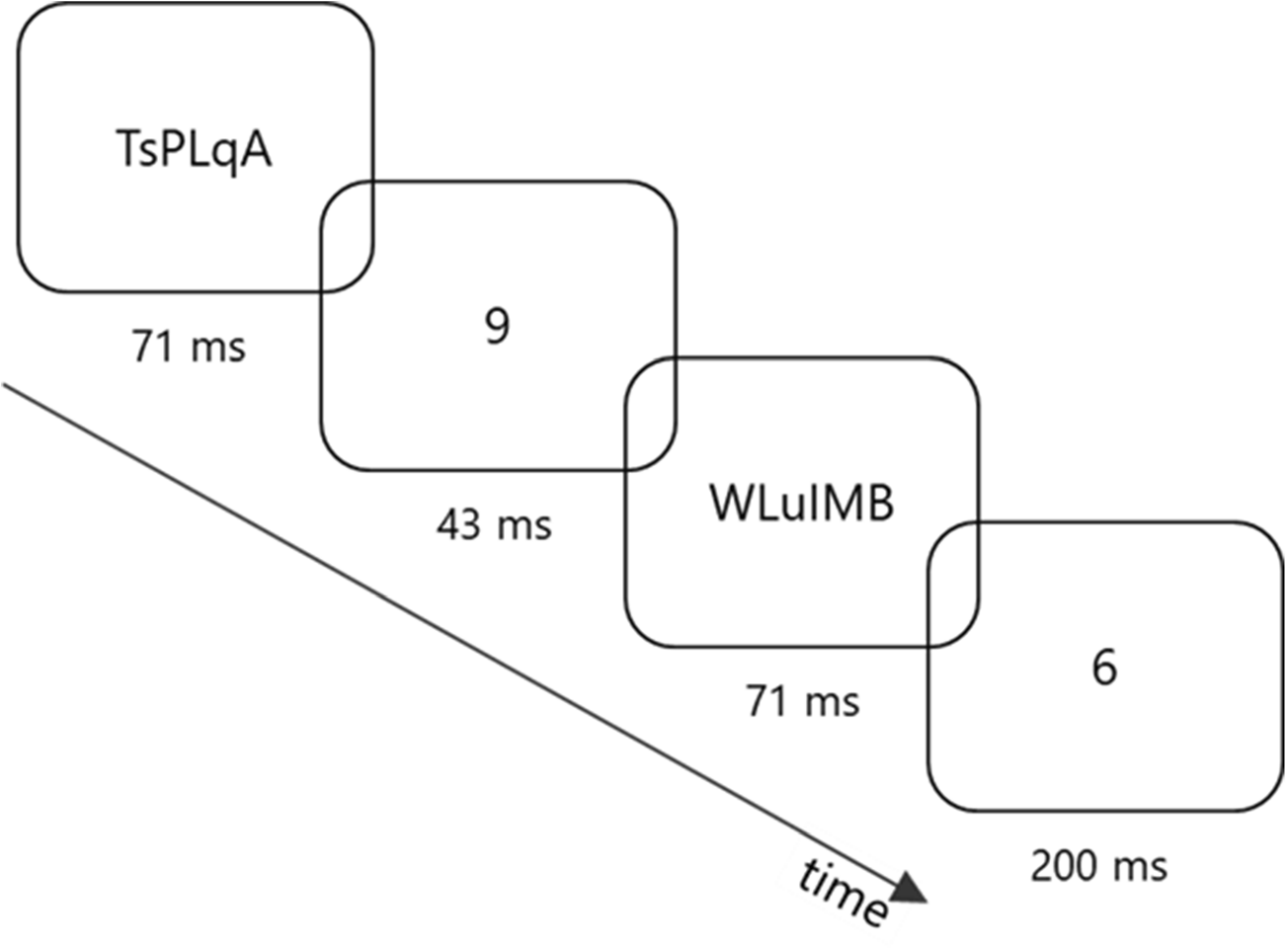
Example stimulus sequence of current study. Four stimuli (forward mask, prime, backward mask, target) were presented in succession in each trial. Stimuli were presented in white on a dark grey background. Prime and target could either belong to the same category (congruent: e.g., both smaller than 5) as shown in this figure or to different categories (incongruent: e.g., prime larger and target smaller than 5). In Experiments 1 and 3, prime and target consisted of numbers between 1 and 9 either depicted as Arabic digit (e.g., 1) or verbal word (e.g.. EINS). In Experiment 2, only Arabic numbers were used. The tasks were to judge whether the target (indirect task) or the prime (direct task) was smaller or larger than 5. In Experiment 3, timing was slightly different: Masks were presented for 70 ms and prime duration ranged from 10 ms to 80 ms with steps of 10 ms

The stimuli (Fig. 3) were similar to the study of Dehaene et al. (1998). All stimuli were visual stimuli displayed at the center of the screen (font Courier New, font size 36 pixels and bold text). Prime and target stimuli consisted of numerals out of 1, 4, 6, or 9 either depicted as Arabic numbers (1, 4, 6, or 9) or as German verbal numbers (EINS, VIER, SECHS, or NEUN). Hence, the stimulus set consisted of 64 prime-target pairs. Before and after the prime, a mask stimulus was presented. Masks were composed of seven randomly drawn characters from {a-z, A-Z}. All stimuli were presented in white (84 cd/m^2^) on a dark gray background (0.1 cd/m^2^).

### Procedure

The experiment consisted of two tasks, with each task being divided into four experimental blocks preceded by one practice block. A practice block spanned 16 trials, which were not considered in the analysis. Each experimental block comprised 64 trials, leading to 256 trials per participant and per task. Each participant conducted both tasks in succession: First the indirect task and then the direct task.

Participants responded with their left or right index finger by pressing a left or right response-button. In both tasks, they performed a simple semantic categorization: In the indirect task, they pressed left or right as quickly as possible according to the target stimulus being larger or smaller than 5. In the direct task, they pressed left or right (without time-pressure) according to the prime stimulus being larger or smaller than 5. The assignment of the response buttons was counterbalanced across participants. Before the beginning of the direct task, participants were informed about the presence of the prime and the stimulus sequence.

The stimulus sequence was the same for all tasks: Each trial started with the display of a fixation cross (420 ms) in the center of the screen. Right after, forward mask (67ms), prime (42 ms), backward mask (67 ms), and target (200 ms) were presented. The stimuli and the presentation thereof (i.e., the trial structure) were the same for all tasks, except for masks (which were generated anew for each forward and backward mask) and the order of the prime-target combinations within an experimental block (randomized anew for each block).

### Data analysis

For the indirect task, RT was defined as the time between the onset of the target stimulus and the key press of the participant (indirect measure). The indirect task was analyzed in three metrics: millisecond (ms), accuracy (% correct), and *d’*. We converted the continuous indirect measures (in ms) into *d’* for each participant by the median-split technique, as described above. RTs shorter than the median were classified as corresponding to a congruent trial, while RTs longer than the median were classified as incongruent. Classification accuracy was then calculated by evaluating these classifications relative to the true condition (congruent or incongruent). The classification performance in the direct task was measured in accuracy (% correct) and *d’*. Response accuracy (% correct) was calculated by taking the percentage of correct answers in proportion to the total of all responses of that participant. The sensitivity *d’* was calculated by defining one type of prime (i.e., primes being > 5) as the signal. Then, trials in which participants responded to the prime as being > 5 when the prime was actually > 5 were counted as hits and trials with responses > 5 when the prime was actually < 5 as false alarms.

Trials with wrong responses in the indirect task or RTs exceeding a certain time frame (indirect task: RT < 100 ms or RT > 1,000 ms; direct task: RT < 100 ms or RT > 5,000 ms) were excluded and the corresponding trial type was assigned back to the pool of trials that still were to be performed and then randomly selected again at a later time during the same experimental block. This kept the final number of trials per block constant.

Values are presented as mean ± between-subjects SEM. A significance level of α = 0.05 was used for all statistical tests. All tests were performed two-tailed to also prove the opposite of an ITA (i.e., a direct task advantage). Importantly, the results did not change when testing one-tailed. Because for some statistical tests sphericity was violated, all *p-*values are reported as Greenhouse-Geisser-corrected values (Greenhouse & Geisser, 1959).

### Comparison with original studies

**Fig. 4:**
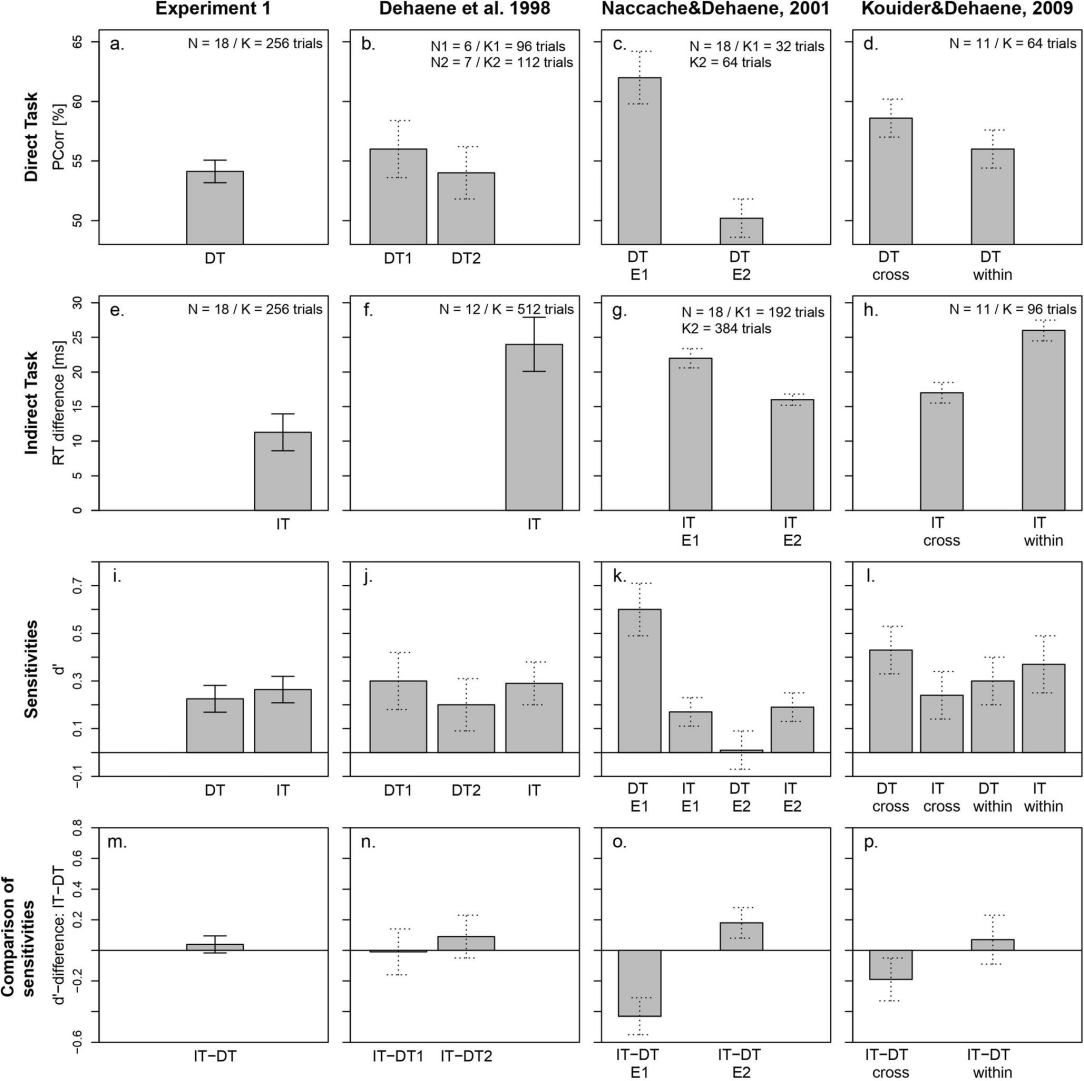
Comparison of our Experiment 1 to the original studies. Columns represent the different experiments of the different studies and rows represent the results from the direct task (DT), the indirect task (IT) according to the standard analysis (***a–h***), and the corresponding sensitivities with the comparison of sensitivities (IT-DT) according to the sensitivity analysis (***i–p***). (***a–d***) Average percentage of correctly classified primes in the direct task. (***e–h***) Mean reaction time (RT) difference (priming effect) between congruent and incongruent trials in the indirect task. (***i–l***) The direct and the indirect task performances using the sensitivity d’. For the indirect tasks from the original studies, the sensitivities were estimated according to the reanalysis proposed by Meyen et al. assuming a conservative q^2^ of 0.0225 (cf. benefit-of-doubt approach; Meyen et al., in press). (***m*–*p***) Difference in sensitivities between the indirect and the direct tasks. An ITA would mean that the sensitivity in the indirect task is significantly larger than the sensitivity in the direct task. This was not the case in any of the studies. Overall, our results from Experiment 1 fit quite well with the literature and show that the original effects are stable and replicable effects. For the study by Kouider and Dehaene (2009), we only report their first experiment. N = total number of participants, K = total number of trials, E1 = Experiment 1, E2 = Experiment 2, cross = masked cross-notation trials, within = masked within-notation trials. Error bars represent between-subject standard error of the mean (SEM). We estimated the mean sensitivities in the indirect tasks and standard errors for the original studies (dashed error bars) according to the reanalysis proposed by Meyen et al. (in press) assuming a q^2^ of 0.0225. For details on the selected experiments and extracted values see our *Methods* section of Experiment 1 and Details on selected values from original studies

Before we present our results, we sketch the conditions and experiments of the original studies that we used to compare to the results of our Experiment 1 (cf. Fig. 4). We extracted the values reported in the original studies and estimated additional values (e.g., sensitivities for the indirect tasks) by using the ITA calculator provided by Meyen et al. (in press; for more details see Details on selected values from original studies).

For Dehaene et al. (1998), we used their behavioral data. From Naccache and Dehaene (2001a), we chose the results for old set primes in their Experiment 1 and the results for both old and new set primes combined in Experiment 2. In the study of Kouider and Dehaene (2009, their Experiment 1), results are reported separately for “within-notation” trials (same prime and target notation) as well as “cross-notation” trials (different prime and target notations). Therefore, we used the priming effects and direct task sensitivities for the masked within-notation and masked cross-notation conditions. We only report the results from their Experiment 1 because it is closest to our Experiment 1 and to Dehaene et al. (1998).

### Results

In Fig. 4, comparison of the results from Experiment 1 with the results of the original studies are shown for the standard analysis (Fig. 4a–h) and for the sensitivity analysis (Fig. 4i–p).

#### Direct measures: prime identification performance

In the direct task, participants classified the prime correctly in 54.1% (*SD* = 4.0) of the trials (t-test against 50%: *t*(17) = 4.3, *p* < .001; see Fig. 4a), indicating above-chance performance with a sensitivity of *d’* = 0.23, *t*(17) = 4.0, *p* < .001 (Fig. 4i) according to the standard analysis.

#### Indirect measures: priming effects

Figure 4e shows the priming effect according to the standard analysis. RTs in congruent trials were on average shorter than RTs in incongruent trials (difference *M* = 11.3 ms, *SD* = 11.3, *t*(17) = 4.2, *p* < .001). A similar pattern has been shown in the original studies (Fig. 4f–h).

The sensitivity analysis resulted in a sensitivity of *d’* = 0.27, *t*(17) = 4.8, *p* < .001 (Fig. 4i), which corresponds to 55.3% (*SD* = 4.6) correctly classified trials using the median-split technique.

**Fig. 5:**
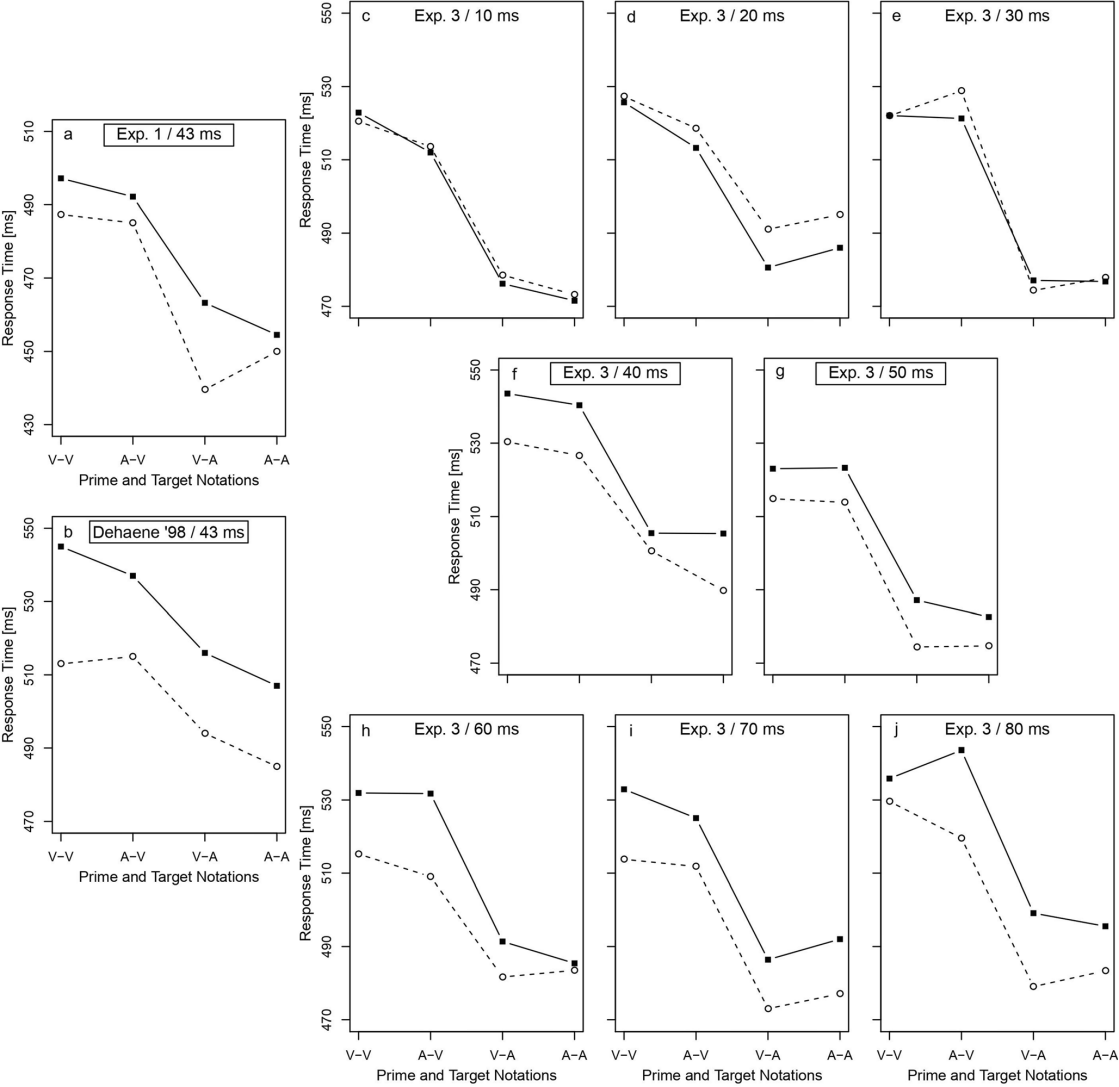
For Experiments 1 and 3 and the original study of Dehaene et al. (1998), average correct response times (RTs) on the indirect task are shown as functions of prime-target congruency (congruent and incongruent) for different prime and target notations (V, verbal; A, Arabic). All three experiments show a very consistent pattern of the RTs between the different conditions V-V, A-V, V-A and A-A. (**a**) In Experiment 1, the mean RTs in congruent trials were systematically shorter than the mean RTs in incongruent trials. (**b**) In the original study by Dehaene et al. (1998), a similar pattern of RTs can be observed. (**c–j**) For Experiment 3, RTs on the indirect task at eight prime durations (ranging from 10 ms to 80 ms by 10 ms) are shown. The RT difference between congruent and incongruent conditions increased with increasing prime duration. The 40-ms and 50-ms conditions had a similar prime duration compared with the conditions tested in Dehaene et al. and Experiment 1. In all three experiments, there is a downwards trend of RTs from trials with V-V notation to trials with A-A notation underlining the consistency of the results. Values for Dehaene et al. (1998) were digitized from the original figure (their Fig. 2a) and plotted anew

Figure 5a shows the mean RTs on the indirect task for the different prime and target notations with a similar pattern compared to the original study of Dehaene et al. (1998). In accordance with their study, congruent RTs were systematically shorter than incongruent ones (Fig. 5b).

#### Direct versus indirect performance (ITA)

To test for an ITA, we compared the direct and the indirect measures of the two tasks using the sensitivity *d’*. The sensitivity in the indirect measure did not differ significantly from the sensitivity in the direct measure (difference: *M* = 0.04, *t*(17) = 0.7, *p* = .5; Fig. 4m).

### Discussion

In Experiment 1, we successfully replicated the results from the original studies. In the direct task, participants performed close to chance level with 54% correct (corresponding to *d’* = 0.23). This performance is almost identical to the direct task performance in Dehaene et al. (1998), who reported 56% correct and 54% correct in their two direct tasks (corresponding to *d’* = 0.3 and *d’* = 0.2, respectively).

In the indirect task, we found that the RTs in congruent trials were on average shorter than the RTs in incongruent trials, which is consistent with the literature (Fig. 4e–h). Also, we found a consistent pattern of results between our Experiment 1 and Dehaene et al. (1998) for the different prime and target notations (Fig. 5a–b). Participants responded faster in trials with Arabic prime-target notations compared to trials with verbal prime-target notations. Overall, this shows that the effects of the original studies are clearly replicable and that there is no doubt about the existence of number priming in the response-priming paradigm.

Most importantly, when focusing on the sensitivity analysis to test for an ITA, we did not find a higher indirect as compared to a direct sensitivity (Fig. 4m; sensitivity difference ∆*d’* = 0.04). That is, we did not find an ITA. Again, this is consistent with the estimated sensitivities we calculated from the literature (Fig. 4m–p). The sensitivities in our Experiment 1, Dehaene et al. (1998), and Kouider and Dehaene (2009) were quite comparable, while the indirect task sensitivities in the study of Naccache and Dehaene (2001a) were even smaller (their Experiment 1: *d’* = 0.17 and Experiment 2: *d’* = 0.19) than the indirect task sensitivities in our Experiment 1 (*d’* = 0.27) and Dehaene et al. (1998, *d’* = 0.29).

Despite the similarities between our Experiment 1 and the original studies, there might be two possible confounds we want to discuss briefly. First, one might be concerned about the different priming effects between the studies. Possible impacts on the size of a priming effect could be physical stimulus properties such as stimulus size or contrast. To test a broader range of stimulus perceptibility, we therefore conducted two more experiments with parametric variations of stimulus contrast and duration. We show in Experiments 2 and 3 that such differences in stimulus properties do not change the overall results.

Second, one could object that the direct task sensitivity was significantly different from zero in our Experiment 1. However, in our direct task, participants classified the masked stimuli with 54.1% correct (Fig. 4a), corresponding to a *d’* = 0.23 (Fig. 4g), which matches very well the discrimination performance in the study of Dehaene et al. (task 1: 56% correct and *d’* = 0.3; task 2: 54% correct and *d’* = 0.2) as well as that of Meyen et al. (54.9% correct and *d’* = 0.25). With regard to the fact that our direct task sensitivity deviated significantly from zero (while that was not the case in Dehaene et al., 1998), there is one important point to make here. It is well known that significance testing is strongly influenced by different factors such as sample size or number of trials (Vadillo et al., 2016). Hence, the fact that the results from Dehaene et al. (1998) were not significantly above chance level can be attributed to the small test power due to small sample size (number of participants N = 6, number of trials K = 96 in task 1 and N = 7, K = 112 in task 2) compared to N = 18 participants, K = 256 trials in our experiment. It is a priori to be expected, that – even if we measured the exact same effect – our results will more likely be significant. Therefore, the focus has to be on the size of the effect (i.e., percent correct or sensitivity) rather than on the significance of the effect alone (see, e.g., Cumming, 2014; Wilkinson & The APA Task Force on Statistical Inference, 1999).

A similar problem arises in the study of Naccache and Dehaene (2001a). In their experiments, the direct tasks (N = 18 participants, K_1_ = 32 trials, K_2_ = 64 trials) were clearly underpowered as compared to the indirect tasks (N = 18 participants, K_1_ = 192 trials, K_2_ = 384 trials) and, therefore, they were more likely to result in erroneous conclusions (i.e., a non-significant result in the direct task; see Vadillo et al., 2016). Again, it is most appropriate to focus on the direct comparison of sensitivities between both tasks rather than to aim for a non-significant result in the direct task.

This highlights the validity of the sensitivity analysis: One cannot rely on significant versus non-significant results but instead should to test for the difference, use the same amount of trials in both tasks, and apply the same metric (i.e., the sensitivity *d‘*). In this context one might also raise the question of whether the direct task used in Experiment 1 is the most appropriate one when comparing direct and indirect measures. For Experiment 1, we wanted to be as close as possible to the literature and, therefore, we used the same direct task as the original studies. In Experiment 3, we introduce a variant for the direct task and show that the overall results did not change.

To summarize, when comparing the direct and indirect tasks using the sensitivity analysis in our Experiment 1 (Fig. 4m), no significant difference in sensitivity and, therefore, no evidence for an ITA was found. A similar pattern appeared in the original studies (Fig. 4m–p) when using the reanalysis proposed by Meyen et al. (in press). In the study of Naccache and Dehaene (2001), the results from their Experiment 1 even showed the opposite of an ITA.

## Experiment 2: Variation of prime contrast

In Experiment 1 we did not find a higher sensitivity in the indirect task as compared to the direct task. That is, there was no ITA. Albeit all stimulus parameters had been chosen to be as close as possible to the original studies, it could nevertheless be conceivable that we had missed the critical range of values where an ITA might show up. Therefore, we now varied the stimulus parameters of the prime over a wide range of values. That is, we chose the stimulus

parameters of the prime such that the sensitivity to the prime in both tasks was varied from low to high. This was achieved by varying the contrast of the prime in Experiment 2 and the duration of the prime in Experiment 3. This parametric variation of the stimulus properties (for similar approaches see F. Schmidt et al., 2011, and T. Schmidt & Vorberg, 2006) increased our chances to find an ITA if there is a critical range where an ITA shows up.

### Participants

Twenty participants (14 female and six male) from the same population and with the same characteristics as in Experiment 1 took part in Experiment 2. Seventeen participants were right-handed by self-report. The age of the participants ranged from 19 to 38 years (*M*_age_ = 23.6, *SD*_age_ = 4.5).

#### Sample size and power analysis

To determine our sample size of N = 20 participants, we used the same approach as in Experiment 1. To compensate for the additional variation of prime contrast, we increased the number of trials and participants for Experiment 2 but we could not measure as many trials for each individual contrast level as in Experiment 1 without prolonging the experiment drastically and thereby tiring the participants. Therefore, the power for each individual contrast level is smaller than in Experiment 1. However, measuring one single data point as precisely as possible was the aim of Experiment 1 but not of Experiment 2. The focus of Experiment 2 was on the parametric variation of the sensitivities and observation of the changes in both tasks. Therefore, we tried to not prolong the experiment too much on the one hand (i.e., the number of trials) and on the other hand to have a reasonable number of trials to estimate the sensitivities at each contrast level (i.e., reasonable power). Again, we used Meyen et al. (in press) to estimate the variability of the congruency and ITA effects. For the congruency effect at a single level of prime contrast, this resulted in a power of 80% for an RT difference of 12 ms (with K = 48 trials, SD = 21.6). For the ITA effect at a single level of prime contrast, the power for an ITA of ∆*d’* = 0.25 was 60.6% and the power for an ITA of ∆*d’* = 0.51 was 98.8%.

Note that these power values are only the minimally achieved powers when assuming the worst-case scenario, in which an ITA would only appear at one single contrast level (but not at the adjacent contrast levels). However, we would expect the indirect sensitivity curve to be above the direct sensitivity curve for more than one single contrast level if the indirect task sensitivity at some point exceeds the direct task sensitivity, because we measured adjacent contrast levels. Therefore, the power to detect an ITA over more than one contrast level was higher than calculated above (due to an increased number of trials when merging adjacent datapoints).

### Setup and stimuli

The experimental setup and stimuli were similar to those described in Experiment 1 except for the following changes: The prime was presented at eight different contrast levels (luminances were 0.3 cd/m^2^, 0.7 cd/m^2^, 1.4 cd/m^2^, 2.9 cd/m^2^, 4.9 cd/m^2^, 7.8 cd/m^2^, 11.6 cd/m^2^, and 16.4 cd/m^2^ vs. background with 0.1 cd/m^2^). All other stimuli (fixation cross, masks, and targets) had a luminance of 4.9 cd/m^2^. Prime contrast levels were chosen using a correction function based on the psychometric brightness function (Schumann & Müller, 2013) to have *n* linearly perceived brightness differences between the brightness conditions *Y*_*i*_ (in rgb ranging from rgb = [20, 110]):

$$ {Y}_i={Y}_0+{\left(\frac{i}{n}\right)}^{\frac{\nu }{\gamma }}\times \left({Y}_n-{Y}_0\right) $$

where ν = 3 is a constant to correct for the non-linear perception of brightness (Stevens, 1957) and γ = 2.2 is the gamma value of the monitor. The brightness condition *Y*_*0*_ = 14.39 can be calculated by taking the lowest and highest brightness *Y*_*1*_ = 20 rgb and *Y*_*n*_ *=* 110 rgb. Contrast of the stimuli was measured using a colorimeter (CS-100A, Konica Minolta Sensing Europe B.V., Netherlands). Only numeral digits (1, 4, 6, or 9) were used as primes and targets to achieve a higher test power for the effect of prime contrast on both tasks.

### Procedure and data analysis

The procedure and data analysis were similar to Experiment 1, except for the following changes: The experiment consisted of two tasks with each task being divided into three experimental blocks preceded by one practice block. Each experimental block comprised 128 trials leading to K = 384 trials per participant and per task.

Prime contrast was counterbalanced within each experimental block. In the direct task, we used a confidence scale as a continuous measure. The scale ranged from -100 to 100 and was presented as a slider. Participants were instructed to continuously press the left button for primes smaller than 5 (and vice versa) to move the slider to the left (or right) side of the screen to the desired level. Thereby, extreme values (-100 or 100) indicated that the participants were most confident about their response (i.e., 100 means very confident that the prime was smaller than 5 and -100 means very confident that the prime was larger than 5, and vice versa). The assignment of button presses was counterbalanced across participants. For the current study, we were only interested in the correctness of the response. Therefore, we only analyzed whether participants pressed the left or the right button. The data from the slider scale are not discussed here, but they reveal similar results when applying the sensitivity analysis (no evidence for an ITA).

### Results

**Fig. 6:**
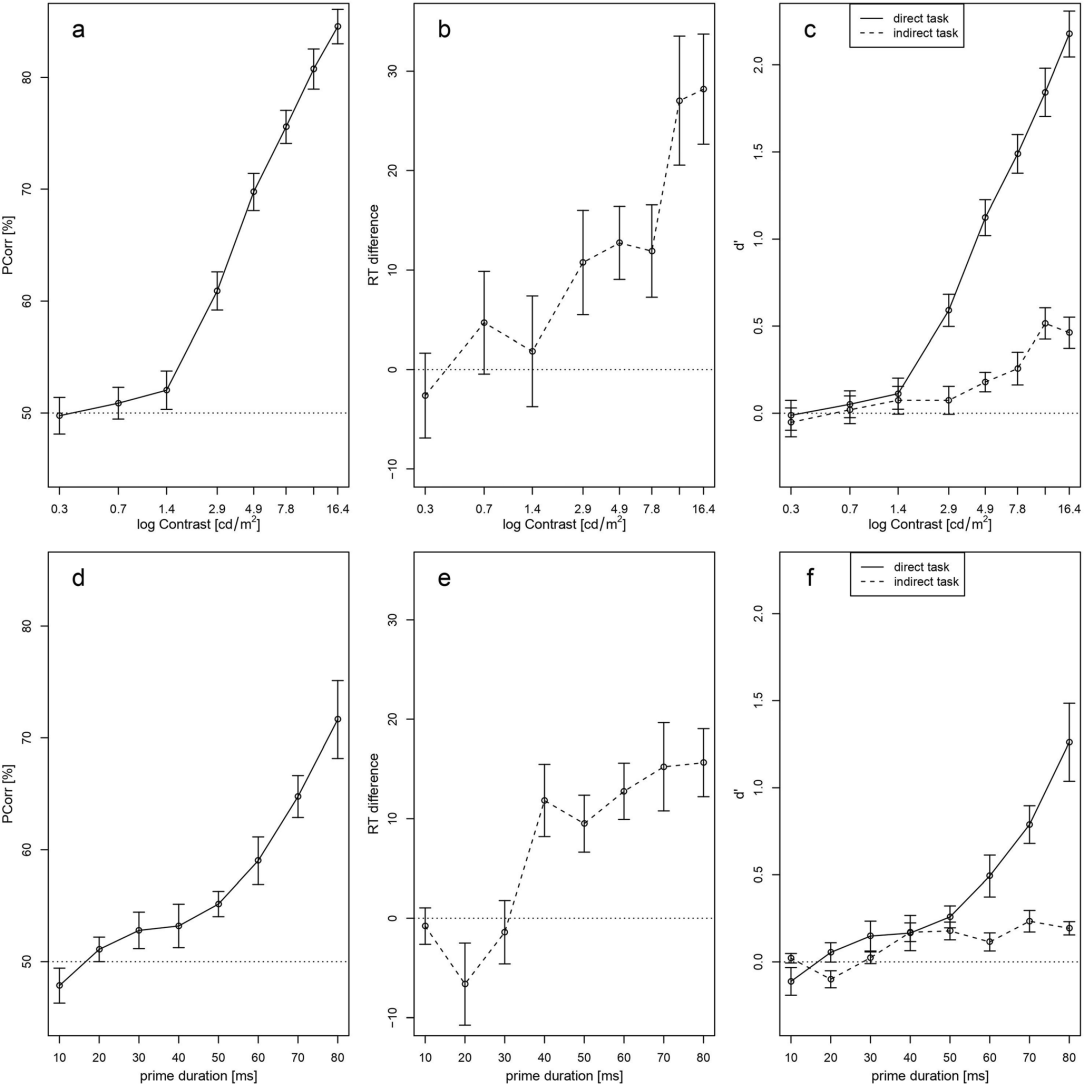
Overview of Experiments 2 (a-c) and 3 (d-f) of the current study. The rows represent the different experiments and the columns represent the results derived from the standard analysis (first and second column) as well as from the sensitivity analysis (third column). The overall patterns show that the prime visibility modulated both tasks. In the direct task (solid lines), percent correct increased with increasing prime contrast (**a**) as well as with increasing prime duration (**d**). A similar pattern appeared in the indirect task (dashed lines; **b+e**). When comparing the two tasks directly with each other by using the sensitivity d’, the indirect task performance did not exceed the direct task performance (**c+f**). Therefore, we concluded no ITA and further, no unconscious processing of numbers. Error bars represent between-subject standard error of the mean (SEM)

The results of Experiment 2 are shown in Fig. 6a–c. Following the standard reasoning, we calculated the percentage of correctly classified primes in the direct task (Fig. 6a) and the RT difference between congruent and incongruent trials (priming effect) in the indirect task (Fig. 6b). Figure 6c shows the sensitivities *d’* for both tasks for each prime contrast, as needed for the sensitivity analysis.

#### Direct measures: prime identification performance

Figure 6a shows the percentage of correctly classified primes on the direct task. Prime identification increased with prime contrast and significantly deviated from chance performance (50%) at a prime contrast of 2.9 cd/m^2^ (*M* = 60.9, *SD* = 7.9, *t*(19) = 6.2, *p* < .001 and higher.

Also, the sensitivity *d’* significantly deviated from chance (zero) at 2.9 cd/m^2^ (*M* = 0.59, *SD* = 0.43), *t*(19) = 6.2, *p* < .001 and higher. Table 1 summarizes the rates of hits and false alarms of classification performance in the direct task.

**Table 1:** Experiment 2: Hits, false alarms, and correct responses

	Prime contrast [cd/m^2^]							
	0.3	0.7	1.4	2.9	4.9	7.8	11.6	16.4
Indirect Task								
Hits	49.0	50.4	51.5	51.3	53.5	55.0	60.0	59.0
False Alarms	51.0	49.6	48.5	48.8	46.5	45.0	40.0	41.0
Percent Correct	49.0	50.4	51.5	51.3	53.5	55.0	60.0	59.0
Direct Task								
Hits	50.7	52.8	50.5	54.2	65.5	71.3	77.5	83.6
False Alarms	50.0	48.4	45.4	35.6	25.9	19.0	15.3	13.4
Percent Correct	49.8	50.9	52.0	60.9	69.8	75.6	80.7	84.5

*Note.* For the indirect task, we applied the median-split technique to determine the response outcomes (hits, misses, false alarms, and correct rejections) from reaction times (RTs). The response outcomes allow to compute the percentage of correctly classified primes (percent correct) and the sensitivity d’ in the indirect task. Here, we show percent correct to give a more intuitive estimation of the task performance. Hits and percent correct are identical at all prime parameter levels since the median as a neutral criterion was selected for each participant

#### Indirect measures: priming effects

The standard analysis yielded a significant priming effect from 4.9 cd/m^2^ (*M* = 12.7, *SD* = 16.4, *t*(19) = 3.5, *p* = .003) upwards. Figure 6b shows the mean RT differences between congruent and incongruent trials at the eight prime contrast levels of the indirect task. On all contrast levels the congruent RTs were systematically shorter than incongruent ones except for the lowest prime contrast (0.3 cd/m^2^).

A repeated-measures ANOVA with the within-subject factors contrast and congruency revealed a main effect of congruency (*F*(1,19) = 57.7, *p* < .001) and an interaction effect of contrast and congruency (*F*(1,19) = 21.7, *p* < .001). Congruent RTs decreased with higher prime contrast whereas incongruent RTs increased.

The sensitivity *d’* significantly deviated from zero at 4.9 cd/m^2^ (*d’* = 0.18, *SD* = 0.25, *t*(19) = 3.2, *p* = .005; see Fig. 6c) and higher. Table 1 summarizes the rates of hits and false alarms of RTs according to the median-split technique.

#### Direct versus indirect performance (ITA)

To test for an ITA, we compared both measures for each prime contrast level directly with each other using the sensitivity *d’.* As shown in Fig. 6c, the sensitivity in the indirect measure did not exceed the sensitivity in the direct measure. At 4.9 cd/m^2^, where the indirect measure deviated for the first time from chance, we found a significant difference in sensitivity with a better direct classification performance (direct: *M* = 1.15, *SD* = 0.47, indirect: *M* = 0.18, *SD* = 0.25), *t*(19) = 6.18, *p* < .001. That is, we found the opposite of an ITA.

### Discussion

The sensitivity analysis has shown that the prime contrast variation did not result in an ITA for either contrast level. That is, the sensitivity in the indirect task did not exceed the sensitivity in the direct task. It was rather a direct task advantage (better sensitivity in the direct task) than an ITA. Especially with low prime contrast, the sensitivity to the prime in the indirect task was just as bad as the sensitivity in the direct task.

It is notable that the increase of the direct task sensitivity (Fig. 6a) was quite steep. This could be due to the within-block variation of prime contrast. Thereby, trials with more visible (i.e., higher contrast) and less visible (i.e., low contrast) primes were intermixed. In line with previous findings (Lin & Murray, 2014; Pratte & Rouder, 2009), these mixed blocks elicited a higher detection rate for the less visible primes as compared to the detection rate for less visible primes in pure blocks (i.e., blocks with only one single prime parameter).

In conclusion, we found that sensitivities in both tasks improved with increasing prime contrast in a similar way and there was no difference between the two measures when prime visibility was low. Further, with higher prime contrast, the improvement was more pronounced in the direct as compared to the indirect measure.

While in Experiment 2 we focused on prime contrast as a means to vary stimulus visibility and the priming effect, many researchers use other methods to achieve different levels of task performance (e.g., variations of SOAs or prime durations; Daza et al., 2002; Dehaene et al., 1998; T. Schmidt, 2002; Vorberg et al., 2003). Thus, we conducted a third experiment in which we also varied prime durations to test a wider range of stimulus perceptibility.

## Experiment 3: Variation of prime duration

Whereas in Experiment 2 we varied prime contrast, in Experiment 3 we chose an alternative method to test different levels of task performance: The variation of prime duration. Thus, Experiment 3 comprises eight groups with prime duration as between-subjects factor. We modified the prime durations parametrically, which ranged from 10 ms to 80 ms in steps of 10 ms. The aim was to test for an ITA in a parametric variation that was similar to the variation of Dehaene et al. (1998). In two control experiments, they had varied prime duration in the direct task to test prime awareness, but, unfortunately, they did not do a parametric variation on the indirect task.

### Participants

A total of 131 native German speakers participated in the experiment. Three of them had to be excluded. The age of the remaining 128 participants (33 males) ranged from 18 to 38 years (*M*_age_ = 24.0, *SD*_age_ = 4.5). The experiments took place at the University of Hamburg. Participants received either course credits or a payment for their participation. The participants were randomly assigned to one of eight groups. Each group comprised 16 participants.

#### Sample size and power analysis

To determine our sample size, we used the same approach as in Experiments 1 and 2. In Experiment 3, the same issue arose as in Experiment 2: Due to the variation of prime duration we could not measure as many data points for each prime duration as in Experiment 1 without going to a very large sample size. This reduces the power for each individual prime duration. However, this is counterbalanced by the fact that we measured adjacent prime durations and that only under a worst-case scenario is there an ITA at just one single prime duration (but not at the adjacent values). As in Experiment 2, the reported power values correspond to this worst-case scenario.

Again, we used Meyen et al. (in press) to estimate the variabilities. For the congruency effect our sample sizes of at least N = 16 participants per single prime duration resulted in a power of 99.4% for an RT difference of 12 ms (with SD = 11.83). For a sensitivity difference of ∆*d’* = 0.25, the power for a single prime duration was 79.9% and for the more substantive sensitivity difference of ∆*d’* = 0.51 the analysis resulted in a power of >99.9%. Unfortunately, some participants had to be excluded due to technical difficulties (see *Data analysis*) such that we updated our power calculations to reflect those (slightly smaller) sample sizes. Assuming the worst-case scenario and N = 11 participants, this resulted in a power of 95.7% for an RT difference of 12 ms (with SD = 11.83), a power of 64.3% for a sensitivity difference of ∆*d’* = 0.25 for one single prime duration, and a power of 99.3% for the more substantive sensitivity difference of ∆*d’* = 0.51.

### Setup and stimuli

Stimuli were the same as in Experiment 1. They were displayed on the center of a 17-in. CRT monitor (Fujitsu Siemens 17P4) with the resolution set at 1,024 x 768 pixels. The refresh rate of the monitor was set at 100 Hz. The experiment was programmed in MATLAB (The Mathworks Inc., Natick, MA, USA) using Psychophysics Toolbox (3.0.14, Brainard, 1997; Kleiner, 2010). The viewing distance was approximately 80 cm.

### Procedure and data analysis

**Fig. 7:**
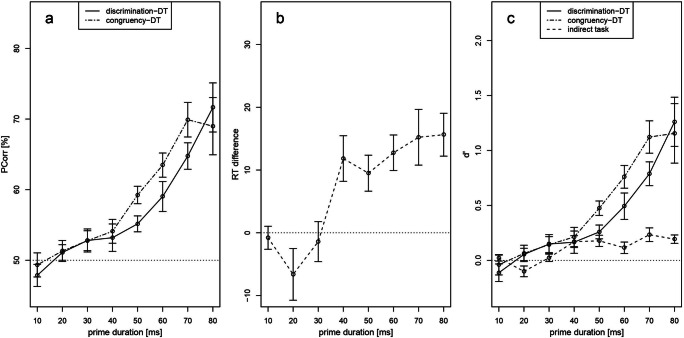
Results from Experiment 3 according to the standard analysis (**a–b**) and the sensitivity analysis (**c**). (**a**) Average percentage of correctly classified primes in the discrimination direct task (discrimination-DT, i.e., is the prime smaller or larger than 5?) and the congruency direct task (congruency-DT, i.e., are prime and target congruent or incongruent?). Percent correct increased with increasing prime duration in both direct tasks with the congruency-DT being more sensitive to prime duration. (**b**) Mean reaction time (RT) difference (priming effect) between congruent and incongruent trials in the indirect task. (**c**) Comparison of the indirect task to the direct tasks using the sensitivity *d’*. For higher prime durations, the direct tasks exceeded the indirect task indicating the opposite of an ITA. No condition provided evidence for a better indirect task sensitivity (no evidence for an ITA) independent of the direct task modality

The procedure and data analysis were similar to Experiment 1, except for the following changes: After the indirect task, participants performed two direct tasks in a counterbalanced order. In the “discrimination direct task” they classified the prime as being smaller or larger than 5, similar to our Experiments 1 and 2. In the “congruency direct task” participants judged the congruency of prime and target (i.e., whether prime and target were congruent or incongruent). Results were similar for both direct tasks and, importantly, the patterns of direct and indirect sensitivities were nearly identical showing no evidence for an ITA. For the sake of comparability to our Experiments 1 and 2, we only focus on the discrimination direct task here but the results from the congruency direct task are reported in Fig. 7. Consequently, for our reported main results, the indirect task contained four experimental blocks, whereas participants performed two experimental blocks on the direct task. The assignment of the button presses was counterbalanced between blocks. Thus, the assignment of the keys was changed after half of the experimental trials (i.e., after two blocks in the indirect task and after one block in the direct task). Participants received initial training (16 trials) before each experimental half to practice the assignment of the keys. In the direct task, trials with too short or too long RTs were not repeated (mean number of trials: 123). The trial sequence was the same as in Experiment 1, but with slightly different timings: Fixation cross (500 ms), forward mask (70 ms), prime (between subjects it ranged from 10–80 ms in steps of 10 ms), backward mask (70 ms), and target (200 ms). The prime duration remained the same within each participant group. Consequently, we had eight parametric variations of prime duration for eight participant groups.

In some of the conditions we recorded EEG data and, in addition, tested variants of the direct task. Due to technical problems, this resulted in incomplete datasets. Therefore, we only report complete datasets without EEG and without variants of the direct task (N = 16 for 50 and 70 ms; N = 15 for 40 ms, N = 13 for 10 ms; N = 12 for 30, 60, and 80 ms, and N = 11 for 20 ms).

### Results

Similar to the analyses in Experiments 1 and 2, we report our results from the standard analysis and from the sensitivity analysis (Fig. 6d–f). In particular, the results for 40 ms and 50 ms are reported, because those conditions are most comparable to the original studies.

#### Direct measures: prime identification performance

Figure 6d shows the percentage of correctly classified primes in the direct task. Prime identification increased with prime duration and significantly deviated from chance (50%) at 50 ms (*M* = 55.2%, *SD* = 4.5, *t*(15) = 4.6, *p* < .001). At 40 ms, prime identification was not significant (*M* = 53.2%, *SD* = 7.5, *t*(14) = 1.6, *p* = .12).

The sensitivity *d’* significantly deviated from chance at 50 ms (*M* = 0.26, *SD* = 0.25, *t*(15) = 4.1, *p* < .001; Fig. 6f), but it was not significant at 40 ms (*M* = 0.17, *SD* = 0.39, *t*(14) = 1.6, *p* = .12). Table 2 summarizes the rates of hits and false alarms of the classification responses.

**Table 2:** Experiment 3: Hits, false alarms, and correct responses

	Prime duration [ms]							
	10	20	30	40	50	60	70	80
Indirect Task								
Hits	50.1	48.5	50.5	53.5	53.5	52.7	54.6	55.1
False Alarms	49.9	51.5	49.6	46.5	46.5	47.3	45.4	44.9
Percent Correct	50.1	48.5	50.5	53.5	53.5	52.7	54.6	55.1
Direct Task								
Hits	48.2	49.6	48.8	50.6	49.4	54.4	60.8	71.7
False Alarms	51.3	46.4	44.5	44.8	39.3	36.0	31.3	22.5
Percent Correct	48.5	51.6	52.1	52.9	55.2	59.2	64.8	74.6

*Note.* Same as Table 1, but with different prime durations

#### Indirect measures: priming effects

Figure 5c–j shows the mean RTs in the indirect task for the different prime and target notations at each prime duration. The RT difference between congruent and incongruent trials increased with increasing prime duration and was systematically shorter for congruent trials from 40 ms upwards. The results from the standard analysis are shown in Fig. 6e. The priming effect was significant from 40 ms upwards (at 40 ms: difference *M* = 11.8 ms, *SD* = 14.1, *t*(14) = 3.3, *p* = .006; at 50 ms: difference *M* = 9.5 ms, *SD* = 11.5, *t*(15) = 3.3, *p* = .005).

The sensitivity *d’* significantly deviated from chance at 40 ms and longer (at 40 ms: difference *M* = 0.17, *SD* = 0.21, *t*(14) = 3.2, *p* = .007; at 50 ms: difference *M* = 0.18 ms, *SD* = 0.20, *t*(15) = 3.5, *p* = .003; Fig. 6f). Table 2 summarizes the rates of hits and false alarms of RT responses according to the median-split technique.

#### Direct versus indirect performance (ITA)

To test for an ITA, we compared the sensitivities in the direct and the indirect task for each prime duration directly with each other. As shown in Fig. 6f, the sensitivity in the indirect measure did not exceed the sensitivity in the direct measure. At 40 ms, the sensitivity in the direct task did not significantly differ from the sensitivity in the indirect task (difference *M* = 0.01, *SD* = 0.40, *t*(14) = 0.1, *p* = .96). At 50 ms, we found an even larger sensitivity for the direct as compared to the indirect task (*M* = 0.08, *SD* = 0.37, *t*(15) = 0.9, *p* = 0.4).

### Discussion

Experiment 3 is a perfect example of how the fallacy of the standard reasoning can lead to erroneous conclusions about unconscious processing: First and most important is that with the sensitivity analysis we found no evidence for a higher sensitivity in the indirect as compared to the direct task. That is, we found no evidence for an ITA.

In addition, our data also allow us to again demonstrate why the standard reasoning is problematic: With the sensitivity analysis, at a prime duration of 40 ms the sensitivities did not differ (no ITA). However, the standard reasoning would have mistakenly claimed an ITA: According to the standard analysis, the clear priming effect in the indirect task on the one hand and a non-significant close-to-chance performance in the direct task on the other hand would suggest a higher sensitivity for the prime in the indirect task. This fits well with the interpretation of the results in the original study of Dehaene et al. (1998). The authors also found a significant priming effect at a prime duration of 43 ms and a non-significant close-to-chance performance in the direct task. They applied the standard reasoning and concluded better sensitivity in the indirect task than in the direct task. However, this conclusion is based on an erroneous reasoning (Meyen et al., in press). When the sensitivity analysis was used, we found similar sensitivities in both tasks in our own three experiments, as well as in the experiment of Meyen et al. (in press) and the re-analysis of Dehaene et al. (1998).

The parametric variation of prime duration revealed that the improvement of the sensitivity was even more pronounced in the direct as compared to the indirect measure, which is in line with the results from Experiment 2. This shows why parametric variations are important for testing direct versus indirect sensitivities in the response-priming paradigm.

Overall, the results of Experiment 3 are very consistent with the results of Experiment 1 and the original studies (Fig. 5). In groups with a comparable prime duration (40 ms and 50 ms), mean RTs in congruent trials were systematically shorter than mean RTs in incongruent trials. Also, participants responded faster in trials with Arabic prime-target notations compared to trials with verbal prime-target notations. This again shows that the original priming effects are clearly replicable and that there is no doubt about response priming for numbers. However, the sensitivity analysis contradicts the interpretation of a higher sensitivity in the indirect task as compared to the direct task.

## General discussion

The present study addresses the question of whether there really is preserved unconscious processing in the masked response-priming paradigm, as claimed in many studies (Dehaene et al., 1998; Kouider & Dehaene, 2009; Mattler, 2003; Naccache & Dehaene, 2001a, 2001b; Pessiglione et al., 2007; ten Brinke et al., 2014; Wójcik et al., 2019). As an important test case, we investigated unconscious processing of numbers as claimed in the original studies (Dehaene et al., 1998; Kouider & Dehaene, 2009; Naccache & Dehaene, 2001a). More specifically, we tested whether an indirect task advantage (ITA) for the processing of numbers can be established when applying appropriate statistical methods (Franz & von Luxburg, 2015; Meyen et al., in press).

First, we replicated the findings of the original studies in Experiment 1. Following the standard reasoning, a clear priming effect in the indirect task and a close-to-chance performance in the direct task would imply an ITA, although the sensitivity analysis revealed that there is no ITA. Thus, we found no evidence for unconscious processing because the sensitivity to the primes was equally low in the two tasks.

Second, we applied parametric variations of prime visibility to test a wide range of stimulus perceptibility and to ensure that we would not miss possible conditions with an ITA (Experiments 2 and 3). In both experiments, we found that the sensitivities of the indirect measures did not exceed those of the direct measures. Instead, it was the improvement of the direct measures that was more pronounced than the improvement of the indirect measures. One merit of parametric variations is the wide range of stimulus perceptibility that is tested. Instead of comparing the performance in the direct and indirect task at only one level of perceptibility, researchers can observe and compare the relative changes of the two measures. An ITA is likely to be found if: (a) the changes in one task differ from those in the other task, and (b) those changes constitute an improvement of the indirect measure that is more pronounced than the improvement of the direct measure along the same stimulus variations (T. Schmidt et al., 2006).

In our experiments, the sensitivity in the indirect task did not exceed the sensitivity in the direct task (no evidence for an ITA). Strikingly, for conditions in which the direct task sensitivity was low and close to chance (e.g., Experiment 3 at 40 ms: *d’* = 0.15), the indirect task sensitivity was similar (*d’* = 0.17) and neither measure significantly differed from each other. Therefore, we conclude that there is no evidence for an ITA and, thus, no evidence for unconscious processing of numbers when applying parametric variations and directly comparing the sensitivities in the two tasks.

Is the calculation of the sensitivity in the indirect task fair? One might be concerned about the decreased sensitivity in the indirect task due to the dichotomization of the RTs. But the direct task responses are binary as well. Since participants have a continuous sense about their responses (i.e., confidence; cf. Zehetleitner & Rausch, 2013), the dichotomization in the direct task also discards information (Cohen, 1983). Therefore, the median-split of the RTs

only equates the two tasks (Meyen et al., in press). With the median-split, we used the best possible classification in the indirect task, thereby giving the indirect task the best possible performance and increasing the chances of finding an ITA (cf. benefit-of-the-doubt approach; Meyen et al., in press). Following a suggestion from one of our reviewers, we also tested a shifting-median criterion in Experiment 1 to counteract influences of practice on RTs (i.e., faster RTs with experimental progress). For this, we calculated a “shifting” median of the RTs up to the preceding trial N-1 to classify the RT in a trial N. Overall this did not change the results (overall median-criterion: *d’* = 0.26, *t*(17) = 4.74, *p* < .001 vs. shifting median-criterion: *d’* = 0.24, *t*(17) = 4.27, *p* < .001).

Still, the question remains whether some ITAs claimed in the literature might withstand our critique and under which conditions such ITAs could be found. One possibility would be that ITAs depend on the complexity of stimuli and task settings. In a study by Schmidt (2002), meta-contrast masking was used with color stimuli, which seems to be less complex than, for example, processing the semantic meaning of numbers. It could be possible that simple stimuli (e.g., Gabor patches) with low level visual features might quickly feed through the system (e.g., by fast feedforward processing; Lamme & Roelfsema, 2000; T. Schmidt et al., 2006) and affect higher response stages, whereas more complex stimuli (e.g., the semantic meaning of numbers or the monetary value of coins; Dehaene et al., 1998; Pessiglione et al., 2007) might require more complex processing that prevents such a direct influence on higher response stages (e.g., because they require feedforward as well as feedback processing). This view would be consistent with the results from our present study, where we found no evidence for an ITA using relatively complex stimuli (i.e., number priming). The sensitivity to the masked prime in the indirect task was just as bad as the sensitivity in the direct task, when visibility was low. We found that the direct sensitivity in particular benefits from a higher visibility of the stimulus, suggesting efficient top-down control for the processing of task-relevant stimuli.

Finally, it must be acknowledged that there are other possibilities for measuring indirect effects on behavior such as EEG or fMRI. For example, the lateralized readiness potential (LRP) in EEG has been used to show different response activations of the prime depending on its compatibility with the target (Dehaene et al., 1998; Eimer, 1993, 1995; Jaśkowski et al., 2008; Leuthold & Kopp, 1998). Here, the same standard reasoning has been applied in the past to show an ITA. We argue that for the same reasons, a significant indirect effect on the LRP cannot be interpreted as good sensitivity for the prime but must be transformed into the same metric as the direct task. Measuring event-related brain potentials using EEG could – in contrast to RTs – allow for a better measurement of indirect sensitivity (Hannula et al., 2005), because response activation takes place before motor output. Future research is needed to clarify this question. Furthermore, the generalizability of our analysis to different paradigms and approaches that investigate awareness-independent processing has to be examined.

In closing, we found no evidence for an ITA and, therefore, no evidence for unconscious processing of numbers. This contradicts the interpretation from the original studies and raises the question of whether other studies exist that might have mistakenly concluded an ITA and, consequently, better unconscious processing. To tackle this issue, we have shown that it can be very useful to apply parametric variations in direct and indirect measures and observe their relative changes. Our suggested approach and statistical analyses offer a tool for unconscious priming research that is easy to use, and provides further insights into the mechanisms of direct and indirect visual processing.

## Details on selected values from original studies

For each original study, we sketch the experimental conditions and show which values we extracted (Table 3). Because only the mean sensitivities *d’* in the direct tasks and the mean RT differences (with corresponding F or t values) in the indirect tasks were reported in the original studies, we had to estimate the standard errors and the indirect task sensitivities. Therefore, we used the ITA calculator (http://www.ecogsci.cs.uni-tuebingen.de/ITAcalculator/) provided by Meyen et al. (in press). To estimate the accuracies for the direct tasks, we then transformed the sensitivities *d’* into % correct with the following formula: accuracy ≈ *d’* / 5 + 0.5. Similarly, we estimated the corresponding SEM: *SEM*_%-correct_≈ *SEM*_*d’*_ / 5 (see Appendix D of Meyen et al., in press). For % correct or sensitivity values close to chance level – as it is the case in the examined studies – this approximation closely matches the conventional transformation *d*^′^ = 2Φ^−1^(% − *correct*): Between 50% correct and 60% correct, the approximation is excellent, between 60% correct and 70% correct it is still good. Both formulas assume that participants responded without bias.

For the study by Dehaene et al. (1998), we focused on the behavioral data. The authors conducted two direct tasks and one indirect task. We selected the t value from the priming effect in the indirect task and in the direct tasks, we selected the *d’*-values for the prime duration of 43 ms.

Naccache and Dehaene (2001) conducted two experiments and compared the priming effect of “old set” primes (1, 4, 6, or 9) with the effects of “new set” primes (2, 3, 7, or 8). The direct task in their Experiment 1 only corresponds to old set primes. Therefore, we only chose the F-values for old set primes in their indirect task as well. In their Experiment 2, only the combined effects of old set and new set primes were reported for the direct task. Accordingly, we also used for the indirect task the combined priming effect for both sets.

From Kouider and Dehaene (2009), we chose the masked cross-notation as well as the masked within-notation condition of their Experiment 1.

**Table 3:** Extracted values from original studies

	No. of trials **Indirect Task**	Reported value **Indirect Task**	No. of trials **Direct Task**	Reported value **Direct Task**
Dehaene et al. (1998)	256	*t*(11)=6.16	48 / 56	d'=0.3 / d’=0.2
Naccache & Dehaene (2001)E1 E2	96 192	*F*(17)=13.8 *F*(17)=21.62	16 32	d'=0.6 d’=0.01
Kouider and Dehaene (2009) cross Experiment 1 within	48 48	*F*(10)=11.83 *F*(10)=27.78	32 32	d'=0.43 d’=0.3

*Note.* Number of trials does not reflect the total number of trials but the number of trials per condition (i.e., congruent/incongruent), which is required for the ITA calculator. E1 = Experiment 1, E2 = Experiment 2, cross = masked cross-notation, within = masked within-notation

## Declarations

### Open practices statement

The data and main analyses scripts for all experiments are available at the Open Science Framework (https://osf.io/ezg3k/). None of the experiments were preregistered.
