# Correction to: Number processing outside awareness? Systematically testing sensitivities of direct and indirect measures of consciousness

**DOI:** 10.3758/s13414-021-02332-y

**Published:** 2021-06-27

**Authors:** Iris A. Zerweck, Chung-Shan Kao, Sascha Meyen, Catarina Amado, Martin von Eltz, Maren Klimm, Volker H. Franz

**Affiliations:** 1 Department of Computer Science, Experimental Cognitive Science, University of Tübingen, Sand 6, 72076 Tübingen, Germany; 2 General Psychology, Universität Hamburg, Hamburg, Germany

**Correction to: Atten Percept Psychophys.**

10.3758/s13414-021-02312-2

The originally posted PDF version of the article contained minor copy‐editing mistakes caused by the publisher when the HTML was converted to PDF. The corrected version is available since 28 June 2021.

